# Extracellular Vesicles: Role in Inflammatory Responses and Potential Uses in Vaccination in Cancer and Infectious Diseases

**DOI:** 10.1155/2015/832057

**Published:** 2015-08-25

**Authors:** João Henrique Campos, Rodrigo Pedro Soares, Kleber Ribeiro, André Cronemberger Andrade, Wagner Luiz Batista, Ana Claudia Torrecilhas

**Affiliations:** ^1^Laboratório de Imunologia Celular e Bioquímica de Fungos e Protozoários, Departamento de Ciências Biológicas, Universidade Federal de São Paulo (UNIFESP), Campus Diadema, 09913-030 São Paulo, SP, Brazil; ^2^Laboratório de Parasitologia Celular e Molecular, Centro de Pesquisas René Rachou, Fundação Oswaldo Cruz, 30190-002 Belo Horizonte, MG, Brazil

## Abstract

Almost all cells and organisms release membrane structures containing proteins, lipids, and nucleic acids called extracellular vesicles (EVs), which have a wide range of functions concerning intercellular communication and signaling events. Recently, the characterization and understanding of their biological role have become a main research area due to their potential role in vaccination, as biomarkers antigens, early diagnostic tools, and therapeutic applications. Here, we will overview the recent advances and studies of Evs shed by tumor cells, bacteria, parasites, and fungi, focusing on their inflammatory role and their potential use in vaccination and diagnostic of cancer and infectious diseases.

## 1. Introduction

**Figure 1:**
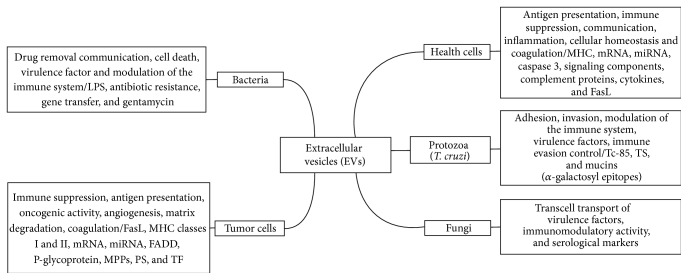
Schematic review of the origin function and markers (molecules to delivery) of EVs normal and tumor cells, parasites, fungi, and bacteria.

Extracellular vesicles (EVs) are particles of 20 nm up to 5 *μ*m in diameter composed of proteins, nucleic acid, and lipids that are found in body fluids such as plasma, serum, saliva, urine, breast milk, ascites, and cerebrospinal fluids [1]. These particles are involved in intercellular communication, modulating a wide range of signaling events during innate and acquired immune responses (Figure 1 and Table 1) [2–4]. EVs are secreted during health conditions or upon inflammation during the course of diseases by all mammalian cells types [2, 3, 5].

EVs include different types of particles and may be named or classified depending on the cell type or function. They can be derived from dendritic cells (dexosomes), prostate tissue (prostasomes), bone, cartilage and atherosclerotic plaques (matrix vesicles), neurons (synaptic vesicles), apoptotic blebs or apoptotic bodies (microparticles, exosomes, and apoptotic vesicles), shed vesicles, shedding microvesicles or microparticles (ectosomes or microvesicles), and membrane fragments of virus infected cells, protozoa, fungi, and bacteria outer membrane vesicles [1, 2, 4, 6–10].

**Figure 2:**
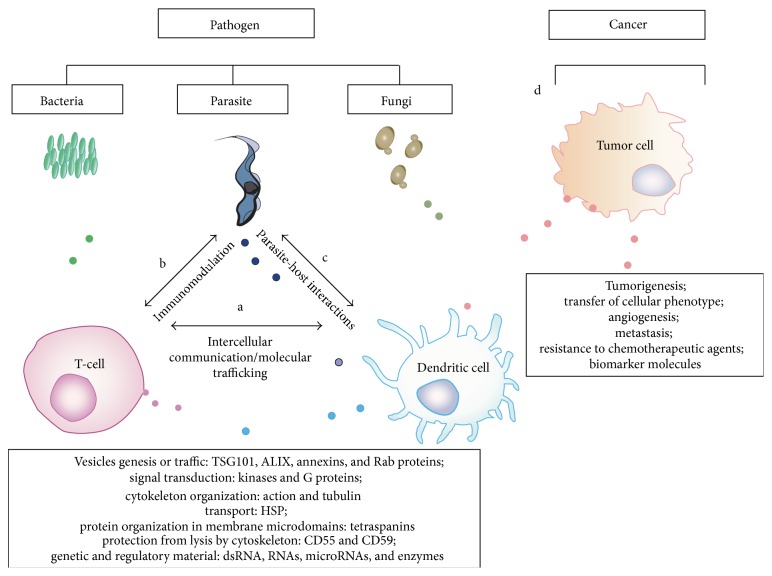
EVs released from all cell types. These particles are involved in physiologic and pathologic processes: (a) intercellular communication and molecular trafficking delivering regulatory signal molecules and (b) and (c) parasite-host interactions and immunomodulation in pathologic conditions; (d) drug resistance, cancer progression, angiogenesis, and metastasis are some functions of exosomes in cancer [1].

The vesicles derived from mammalian cells contain a family of integral membrane proteins that cross four times the lipid bilayer and are called tetraspanins [11], including the surface markers of lymphocytes and antigen-presenting cells such as CD37, CD9, CD53, CD63, CD81, and CD82. EVs also contain molecules of the major histocompatibility complex (MHC classes I and II) (http://www.exocarta.org/) [11, 12]. EVs derived from normal cells cause either suppression or activation of the immune response by modulating the production of inflammatory mediators. For example, T-cells and monocytes secrete vesicles that contain FasL on the surface that modulate apoptosis of the other cells (Figure 2) [13]. Vesicles isolated from monocytes deliver proinflammatory mediators that activate endothelial cells [14, 15]. Tumor cells secrete EVs that are able to downregulate the immune system, allowing the escape from the immune system. Furthermore, these vesicles can control tumor development and growth, by decreasing the expression and release of IL-2 reducing the proliferation of natural killer (NK) cells [14, 15]. Therefore, EVs are potential biomarkers and antigens for vaccination, with potential uses for early diagnostic, and therapeutic applications in several diseases.

The purpose of this review is to provide an updated overview of the vesicles released by distinct pathogens and mammalian tissues, highlighting their potential use in vaccination and diagnostic of cancer and infectious diseases.

## 2. Extracellular Vesicles in Cancer

EVs derived from tumors may be involved in tumor growth control and in the communication events between tumor and normal cells by delivering oncogenic proteins and growth factors [16, 17]. In some cases, EVs suppress tumor growth by exposing dendritic cells MHC classes I or II molecules, peptides, and costimulatory molecules for the immune system. This amplifies the immunological response, preventing tumor growth [18]. EVs can also stimulate the resistance to chemotherapeutic agents. Moreover, EVs contain proteins and genetic material from the originating tumor cells that can be used as diagnostic biomarkers. In this regard, recent efforts to elucidate different roles and signaling pathways of EVs have been conducted.

A pivotal role of EVs during cancer cell migration and invasion has been reported in different cell types. For instance, EVs derived from 786-0 renal tumor cells enhance their migration and invasion properties [23]. This occurs through induction of type 4-chemokine receptor (CXCR4) and matrix metalloproteinase-9 (MMP-9) expression by EVs. In addition, adhesion and invasion of the gastrointestinal interstitial stroma are enhanced by the oncogenic protein tyrosine kinase (KIT) present in tumor cell EVs [46]. More importantly, those structures have also been involved in drug resistance. Tamoxifen-resistant breast tumor cells release exosomes that contain microRNAs (miR221/222) and promote drug resistance in naive cells [19]. Similarly, resistance to docetaxel in breast tumors and prostate cancer, as well as cisplatin in human lung cancer line (A549 cells), was associated with the content of vesicular microRNAs transferred to susceptible cells [47–49]. Moreover, EVs from A549 cells containing TrkB, EGFR, and sortilin receptors (TES complex) were related to angiogenesis induction through endothelial cells [24]. In hepatocellular carcinoma (HCC), one of the most lethal cancers, the tumor becomes more resistant to TGF*β*-dependent chemotherapy through long noncoding RNAs (lncRNAs) obtained from EVs [50]. Therefore, the extracellular communication through EVs is an important mechanism to activate/deactivate certain crucial events in tumor cell biology.

Other studies have evidenced the role of microRNAs present in EVs in cancer establishment. For instance, miR-105, detected in EVs from breast tumor, is associated with metastasis formation via destruction of endothelial monolayers. Interestingly, it is possible to detect miR-105 in the blood circulation before the metastasis establishment reinforcing its potential role as a diagnostic biomarker [20]. Likewise, gastric cancer stromal cells deliver exosomes to gastric tumor cells. Expression of miR-214, miR-221, and miR-222 present in these EVs is related to lymph node metastasis, venous invasion, and tumor development [21]. In some cases, miR-containing EVs repress proangiogenic events and impair tumor development on a bone cancer model [22].

The study of biogenesis of stress-induced vesicles also becomes crucial to understand the development of metastasis. For example, the elevated expression of* RAB22A* gene in breast tumor cells induced by hypoxia, common in advanced tumors, increases the shedding of vesicles, and the Rab protein colocalizes with the sites of budding EVs. Moreover, the knockdown of* RAB22A* prevents metastasis, supporting the idea that Rab is involved in the generation of EVs [25]. EVs released from heat-stressed tumors in a mouse model can induce antitumor immunity [51]. These vesicles showed chemotactic effects on CD4+ and CD8+ T-cells, efficiently activating dendritic cells (DC). Another study showed that EVs derived from breast cancers can alter the tumor microenvironment and promote tumorigenesis of normal cells via induction of autophagy, response to DNA damage repair (DDR), and induction of reactive oxygen species (ROS) in normal breast epithelial cells [52].

EVs also carry potential cancer biomarker molecules, as reported by several groups. This includes the polyadenylate-binding protein 1 (Pabp1), predominant in EVs from metastatic duodenal tumor cell lines [26], prostate-specific membrane antigen (PSA) related to prostate cancer progression, angiogenesis, and metastasis [27], miR-21 and miR-146a in cervical cancer [53], and finally lncRNAs in skin cancer (secreted into the blood or urine through EVs) [54]. All the above-mentioned microRNAs are proposed as potential biomarkers for cancer noninvasive diagnosis. It was also shown that EVs from pancreatic tumor cells contain fragments of double-stranded genomic DNA (dsDNA), suggesting that mutations may be identified in this dsDNA as predictors of cancer and streamline therapeutics [55]. Based on these findings, it is clear that new biomarkers, once optimized, could be used in therapeutic conducts, offering great advantage over other established methods.

In cancer therapy, EVs can also be employed as vehicles to deliver drugs. EVs from tumor cells are able to associate better with their recipient cells than liposomes (>10-fold), due to their lipid and protein composition [56]. In addition, microRNAs can be delivered to tumor cells and interfere with cancer progression and metastasis. In this logic, synthetic miR-143 was introduced into mesenchymal stem cells, and the secreted exosomes containing miR-143 was transferred to osteosarcoma cells to reduce the migration of the latter cell [57]. Interestingly, a feedback regulatory mechanism for controlling exosome release was suggested, in which exosomes derived from normal human mammary epithelial cells could impair the release of exosomes from breast tumor cells [58]. These authors suggest that this may be used as a novel therapeutic approach, attenuating carcinogenic effects of tumor exosomes. Another interesting strategy is to use a synthetic structure based on tumor-derived exosomes and staphylococcal enterotoxin B to induce apoptosis in breast tumor cells [59, 60]. The vesicles could be used as a diagnostic, because tumor cells release vesicles in biologic fluids like urine, blood, ascites, and pleural fluids. For example, patients with ovarian cancer shed vesicles derived from tumor cells in the circulation. These vesicles are enriched up to 4-fold more in patients with cancer than healthy controls. Therefore, they can be used as biomarkers to identify early cancers in asymptomatic patients that will potentially develop malignancy. In addition, specific miRNAs are found in extracellular vesicles from patients with lung cancers [61].

DC have been widely used in the research of therapeutic cancer vaccines. For example, DC were primed with interferon-gamma (IFN-*γ*) to induce the expression CD40, CD80, CD86, and CD54 in exosomes, endowing a potent CD8+ T-cell-triggering potential* in vitro* and* in vivo* [28]. Yao et al. [62] compared the antitumor immunities between EG7 tumor cell-derived exosomes [EXO (EG7)] and EXO- (EG7-) targeted dendritic cells [DC (EXO)]. They showed that the latter DC (EXO) was more effective in inducing antitumor immunity, and this was independent from the host DC, emphasizing the role of the host DC in tumor cell-derived exosomes (TEX) vaccines. In contrast, CD8+ T-cell responses could be induced* in vivo* when mice were immunized with protein-loaded instead of peptide-loaded dexosomes. Recently, protein-loaded dexosomes were used to protect against tumor growth, whereby CD8+ T-cell responses occurred* in vivo* [63, 64].

Purified MHC classes I and II inserted in exosomes and delivered to melanoma were recognized by specific T-cells. This was used to transfer functional MHC/peptide complexes to antigen-presenting cells [65]. In this way, antitumor response could be elicited as these complexes may stimulate CD8+ and CD4+ T-cell responses in an “acellular” immunotherapy approach. In another study, exosomes from Rab27a overexpressing cells increased significantly CD4+ T-cell proliferation* in vitro* because these exosomes upregulated MHC class II, CD80, and CD86 molecules in DC. Moreover, exosomes containing a small GTPase protein involved in secretion of exosomes also were capable of retaining tumor growth* in vivo* [29].

Plasmid DNA vaccines encoding EV-associated antigens were recently used as vaccines in mice in order to produce ovalbumin containing-EV antigens* in vivo*, either exposed on the surface of vesicles or incorporated inside membrane-enclosed virus-like particles [66]. In both cases, these vaccines were able to induce specific T-cell responses and efficiently prevent the growth of ovalbumin-expressing tumors* in vivo*, showing that immunotherapy based on EVs may be a valuable method to promote tumor control and other diseases.

## 3. Bacterial Vesicles

Bacteria release vesicles sizing from 20 to 250 nm [33, 67] are named outer membrane vesicles (OMVs) for Gram-negative and membrane vesicles, or blebs, for Gram-positive bacteria [68, 69]. EVs are required for the exchange of genetic information between bacteria such as* Bacillus anthracis*,* Staphylococcus aureus*,* Mycobacterium ulcerans*,* Bacillus *spp.,* Escherichia coli*,* Pseudomonas aeruginosa*, and* Helicobacter pylori*. Additionally, EVs contain toxins and deliver virulence factors to host cells [32, 70–80]. Bacterial EVs are composed of cytosolic and membrane proteins, lipoproteins, phospholipids, glycolipids, and nucleic acids [31, 32, 81–84]. Detailed composition analysis and biogenesis of OMVs from different Gram-negative bacteria are available [33]. For example, OMVs from* Bordetella parapertussis* contain surface immunogenic molecules, porin, outer membrane protein OmpQ, and pertactin that were used in a murine model to assess the protection against infection [30]. In the same way, OMVs of* Pseudomonas putida* KT2440 have outer membrane proteins such as OprC, OprD, OprE, OprF, OprH, OprG, and OprW which can serve as adjuvants or vaccine [85].* Vibrio cholerae* OMVs contain several proteins that contribute for the virulence and are essential for cell growth and colonization* in vivo* [86]. Another interesting aspect of OMVs is their role in delivering endotoxins to host cells as demonstrated for enterogenic and uropathogenic* Escherichia coli* ((ETEC) and (UPEC)), the causative agents of traveler's diarrhea and human urinary tract infections. Both ETEC and UPEC strains are able to produce many virulence factors including the heat-labile enterotoxin (LT), homologous to cholera toxin, and cytotoxic necrotizing factor type 1 (CNF1). These toxins are released from bacteria in OMVs and delivered to host cells through vesicle internalization [74, 87]. In particular, LT also acts as a ligand for vesicle binding, which is internalized via lipid rafts. Once inside the cell, the toxin is trafficked via retrograde transport through the Golgi and the endoplasmic reticulum [74].

An outstanding role of OMVs in biotechnology is their use as general vehicles to deliver human, heterologous, or viral antigens [33, 88].* Neisseria meningitidis* serogroup B OMVs showed remarkable adjuvant properties for anti-HIV-1 antigens and induced a production of IFN-*γ* and IL-4 [89]. Vesicles isolated from DC infected with* Mycobacterium tuberculosis* were able to induce a protective host immunity response [90, 91]. There are also potential uses of these EVs as cancer vaccines through immune stimulation [92]. OMVs from different species of Gram-negative bacteria contain lipopolysaccharide (LPS), proteins, and nucleic acids, which are strong agonists in the modulation of inflammatory reactions through the activation of Toll-like receptors (TLRs). These activations require the action of LPS, which is sensed by Toll-like receptor 4 (TLR4) on host cells, and induce an innate immune response to Gram-negative bacteria leading to inflammatory cytokine production [93–96].

In the case of* Pseudomonas aeruginosa*, OMVs appear to deliver virulence factors to distant locations by fusing with lipid rafts of several host cell membranes [97]. Proteins present in secreted vesicles released from* P. aeruginosa* also seem to play important roles in pathogenesis. This is the case of the inhibitory factor of the cystic fibrosis transmembrane conductance regulator, which promotes changes in the epithelium, allowing reduced clearance of* P. aeruginosa* toxin A that hijacks the host ubiquitin proteolytic system [97]. Therefore,* P. aeruginosa* EVs have the potential to protect the immunized host against subsequent infection and for this reason they have been proposed as vaccines candidates against infection. Another interesting example is OMVs isolated from* Haemophilus influenzae*, which increases the expression of CD69 and CD86 and activating of the humoral response. In addition, they induce TLR9 signaling through bacterial DNA, which causes a significant proliferative response of inflammatory cells [98].

Vesicles from Gram-negative bacteria are released naturally as blebs of the outer membrane through bulging and “pinching off.” Alternatively, vesicles can be prepared from the detergent-treated bacteria either from normal or from bacteria carrying genetic modifications such as the generalized modules for membrane antigens (GMMA) to induce a strong immune response [99]. All these vesicles are called OMVs, but it is important to note that they have different composition and properties. Naturally shed blebs are almost free of cytoplasmic and inner membrane components and maintain lipophilic proteins, unlike detergent extracted OMVs derived from bacteria. These differences are relevant when considering the use of vesicles for immunization or diagnostic purposes [67]. Several vaccines are prepared based on OMVs isolated from Gram-negative bacteria [100]. One example is the case of* Neisseria meningitidis*-OMVs vaccine, named Bexsero (Novartis) [67]. These particles activate the immune response and protection against a challenge with bacteria in murine models [82, 101–107]. There are, however, several cases that vaccination with OMVs requires further developments to improve better antigenicity, manufacturability, and reduction of pyrogenicity, detergent extract, and toxicity via LPS detoxification [82].

The mechanism of how Gram-negative bacteria-derived OMVs elicit a vaccination effect, for example* E. coli* used as a model to study the effect of the adaptive immune response decrease against bacteria-induced lethality. However, with high doses these OMVs induced systemic inflammatory, characterized by hypothermia, tachypnea, and leukopenia (sepsis) [108].

Because of the thick cell wall of Gram-positive bacteria, extracellular vesicle secretion has been less studied in these bacteria. Nevertheless, it has been reported that* S. aureus* and* Bacillus subtilis* secrete membrane vesicles to the extracellular milieu. Proteomic analysis revealed that vesicles derived from* S. aureus* harbor several pathogenic components [109]. Furthermore,* S. aureus* extracellular vesicles induce atopic dermatitis-like skin inflammation in mice. These observations provided hints on the possible roles of Gram-positive secreted vesicles. Recently, a study on the immune activating role of Gram-positive bacteria-derived EV has been published [110]. The Gram-positive* Bacillus anthracis*, the agent of the Anthrax disease, also shed membrane-derived vesicles. These EVs are formed by a double membrane and have a spherical shape sizing from 50 to 150 nm [83]. They are enriched by molecular chaperons and molecules of the cell wall involved in the cellular architecture and include the lethal toxin (LeTx) and the antholysin (ALO). BALB/c mice immunized with these EVs were able to produce more protective IgM to the toxin in comparison with the isolated toxin, prompting to further use these preparations to elaborate vaccines. The protection induced by vesicles obtained from Gram-positive bacteria was not as effective when compared to Gram-negative bacteria OMVs indicating that further work might be necessary to improve their potential.

In summary, OMVs include multiple virulence factors, overcoming the limitation of a single antigen immunization. Furthermore, OMVs can act as adjuvant and antigen carrier.

## 4. Parasite Vesicles

Cultured protozoan parasites release EVs that contain several molecules that might affect the host (Figure 1). They are composed of membrane fragments and cytosolic components, including proteins, lipids, and nucleic acids that accumulate in the supernatant of the protozoan cultivated in the presence or absence of host cells [40, 41]. When injected in animal models or added to* in vitro* systems, these EVs were found to affect the course of infection and alter the disease progression caused by the parasite, through the modulation of the host innate and acquired immune response. EVs are described in many protozoa such as* Leishmania* spp. [37, 38, 90, 111, 112],* Trypanosoma cruzi* [40, 41, 113–115],* Trypanosoma brucei* [116],* Plasmodium* spp. [117, 118],* Trichomonas vaginalis* [119],* Toxoplasma gondii* [120–122], and* Eimeria* parasites [123]. Helminthes have also released EVs in* Dicrocoelium dendriticum* [124].

*Trypanosoma cruzi* is a flagellate protozoan that causes Chagas disease. It is acquired by humans either by the insect vector, blood transfusion, or through maternal transmission during new born delivery [125]. When* T. cruzi* enters the host, the first line of defense is the innate immune response, which initiates when receptors that recognize microbial products are activated [126]. This occurs through Toll-like receptors (TLR) signaling and macrophage activation by mucin-like glycoproteins, which corresponds to 60–80% of the parasite surface molecules [35, 127], resulting in the increased production of IL-12, IFN-*γ*, and nitric oxide (NO) [128]. The production of these proinflammatory cytokines leads to the activation of several kinds of cells such as natural killer (NK) typical of the acute phase of Chagas disease [129]. Very little is known about how mucin-like glycoproteins and other surface components are presented to the host.

Infective parasites obtained from cultured mammalian cells shed large amounts of EVs that are rich in these surface molecules [40]. EVs isolated from infective* T. cruzi* forms promote macrophage activation with an increase in parasitemia levels and amastigotes nests in the heart tissue [41]. These effects are caused by parasite surface glycoproteins present in the vesicles that attenuate the host immune system.* T. cruzi* EVs are enriched in *α*-gal containing glycoconjugates, found preferentially in the mucin-like molecules [35], and several surface glycoproteins, known as members of a* trans*-sialidase (TS) family that participate in adhesion and invasion of host cells [40, 41, 114, 130]. Mucins containing *α*-gal residues elicit high titers of IgG antibodies decreasing parasitemia during the chronic phase [131]. Therefore, the production and release of EVs might have a key role in the establishment of infection and may be considered a platform to develop preventive or prophylactic vaccines for Chagas disease [132].

*Leishmania* genus encloses protozoan species that cause visceral, cutaneous, and mucocutaneous leishmaniasis in humans. The disease is transmitted by sandfly vectors (*Lutzomyia* and* Phlebotomus*), which inject parasites into the host during the insect blood meal [39]. In culture, the insect stages of several* Leishmania* species release EVs containing parasite antigens, such as the surface glycoprotein of 63 kDa (gp63) that has a strong suppressive effect on host macrophages [37, 38]. However, a missing step in* Leishmania* EVs biogenesis is whether those structures also contain the major surface lipophosphoglycan (LPG), a multivirulence factor involved in the interaction with the vertebrate and invertebrate host [39].

EVs derived from* Leishmania donovani* are involved in immune response evasion mechanisms, enabling parasite survival in the host [37, 133, 134]. In contrast, EVs derived from macrophages infected with* Leishmania amazonensis* induce proinflammatory response* in vitro* by stimulating the production of proinflammatory cytokines TNF-*α*, IL-12, and IL-1*β* [112]. These host-derived vesicles have been characterized and contain both parasite and host components [37, 38], which indicates that a cross talk of signaling events occurs during infection. Indeed, the immunization of mice with dexosomes derived from DC pulsed with* Leishmania major* antigen was able to provide protection against the parasite [90]. This finding could help to improve the available canine vaccines, used to stop transmission [135], and eventually develop a preventive prophylactic human therapy for leishmaniasis.

*Trichomonas vaginalis*, a flagellated protozoan that colonizes human vaginal and urethral epithelia, also secretes vesicles that act at the host-parasite interface.* T. vaginalis* EVs stimulate the immune response by increasing the production of IL-6 and IL-8 [15] and promote greater adherence of less adherent strains of the parasite to the epithelium [15].* T. vaginalis* EVs fuse with and deliver their contents to host cells [136] and are clearly involved in the colonization of the genital host's tract. It is also possible that EVs from this parasite could provide a more suitable environment to other sexually transmitted diseases such as HIV or HPV.

There are several studies about EVs of* Plasmodium* spp., Apicomplexa parasites that cause human and animal malaria focusing mainly in the immunization alternatives. For example, EVs derived from reticulocytes infected with* Plasmodium yoelii*, a rodent malaria, induce protection to infection in mice [137].* Plasmodium berghei*, another rodent malaria, secretes microparticles in the plasma of infected mice that induce an intense macrophage activation, which results in inflammatory reaction [138] via TLR4 and MyD88 [12]. Therefore, these EVs are key components in the modulation and communication between the parasite and the host [118]. However, one of the main difficulties in working with human* Plasmodium*, especially* Plasmodium vivax*, is the availability to have enough amounts of EVs.

*Toxoplasma gondii* is another intracellular Apicomplexa protozoan that causes Toxoplasmosis. The disease is usually transmitted by eating contaminated meat, accidental ingestion of cat feces with oocytes, and congenital contact. It may cause abortion in pregnant women [139]. The infection is severe in immune-compromised individuals. EVs derived from DC incubated with* T. gondii* antigens induce an intense immune response, increasing the levels of MHC class II and the specific production of T-cells and cytokines [140]. Studies of immunization with these DC are promising alternatives in promoting protection against* T. gondii* [141, 142].* Eimeria tenella*,* Eimeria maxima*, and* Eimeria acervulina* are also coccidian parasite of chickens that also release EVs, which confer protective immune response against the parasite [123, 143, 144].

In summary, EVs isolated from several parasites or from infected cells have major effects on the immune response and are also potential candidates for immunoprevention of parasitic diseases.

## 5. Fungal Vesicles

Fungi have the capacity to cause devastating human diseases, some of them with high mortality rates, in both immunocompetent and immunocompromised individuals [145]. Pathogenic fungi exhibit a singular genetic flexibility that facilitates rapid adaptation to the host or environment [146]. However, there are several open questions of how these pathogens colonize and cause morbidity.

As other eukaryotic organisms, fungi use membrane trafficking to connect intracellular and extracellular compartments allowing sorting of protein and lipids to their final cellular sites [147]. For a variety of proteins, the extracellular milieu is the final destination of the cell wall components, digestive enzymes, and, in the pathogenic species, virulence factors [148]. In fungi, the cell wall represents the final step of secretion, an event that brings additional complexity to the secretory mechanisms used by these cells [147]. The cell wall is a complex and rigid structure basically composed of chitin, chitosan, *β*-1,3-glucan, *β*-1,6-glucan, mixed *β*-1,3-/*β*-1,4-glucan, *α*-1,3-glucan, melanin, and glycoproteins as major constituents [149].

EVs are now recognized as important structures for transcell transport of virulence factors that modulate host immune responses [147, 148, 150, 151], suggesting the importance of these structures in the pathogenesis of many fungal diseases. The production of fungal EVs was initially characterized in the pathogenic yeast* Cryptococcus neoformans* [152]. Currently, EVs were identified in several pathogenic fungi such as* Histoplasma capsulatum*,* Paracoccidioides brasiliensis*,* Sporothrix schenckii*,* Candida albicans*,* Candida parapsilosis*,* Malassezia sympodialis* [42, 78, 152, 153], and nonpathogenic yeast* Saccharomyces cerevisiae* [154]. Different proteins, sterols, phospholipids, polysaccharides, and pigments have been characterized in these fungal EVs isolated from culture supernatants [42, 78, 150–158]. Many of these molecules have been identified as known virulence factors or inducers of host humoral responses.

For example, in* C. neoformans* the most important virulence factor and immunomodulator, the glucuronoxylomannan (GXM) [43], was detected in vesicles released during* in vitro* macrophage infection [150]. In* P. brasiliensis*, similar GXM that interacts with *α*1,3-glucans was detected in EVs [159]. GXM acts differently on the host immune response, depending on its specific molecular characteristics [44, 45] making it a possible target for antifungal therapy or vaccination [45].

Another key molecule in fungal infection is glucosylceramide (GlcCer), a glycolipid component of the fungal cell wall [160], which has been detected in EVs of* C. neoformans* [152, 158],* P. brasiliensis* [155], and* C. albicans* [151]. Fungal GlcCer is an antigenic glycosphingolipid that elicits antibody responses in experimental infection models [161] and in patients affected by some mycoses, such as cryptococcosis [162]. GlcCer is described as a virulence regulator of* C. albicans* and* C. neoformans* [163, 164]. Furthermore, GlcCer from* P. brasiliensis*,* Aspergillus fumigatus*, and* S. schenckii* inhibited T-cell proliferation* in vitro* [165]. The GlcCer from* A. fumigatus* was able to activate* in vitro* mouse and human NK cells and to induce airway hyperreactivity in mice [166]. These findings indicate that fungal GlcCer may influence both humoral and cellular responses and that inhibition or blocking the GlcCer action can be a therapeutic approach [160].

Other studies have evidenced that vesicles isolated from* C. neoformans* culture supernatant were able to melanize after incubation with L-DOPA [158], a substrate for melanization [167]. Melanin has been identified in several pathogenic fungi [168]. Although it is immunologically active, little is known about its role in the immune response. Melanin protects fungal cells from phagocytosis by macrophages, a key step in the host defense against these pathogens [169]. It also reduces proinflammatory cytokines [170] and decreases their susceptibility to antifungal drugs [148], mainly to amphotericin B and caspofungin, and is less evident or absent in ketoconazole, fluconazole, or itraconazole [171, 172]. Therefore, it seems that melanization is a distinguished feature observed in EVs released during fungal infections and its role should be further explored in the fungal pathogenesis.

Many studies indicated that acquired immunity against EVs is observed during fungal infections. Vesicular components reacted with immune serum from patients with cryptococcosis, histoplasmosis, and paracoccidioidomycosis (PCM) [42, 78, 153] or with serum from* C. albicans*-infected mice [151]. Particularly, EVs of* P. brasiliensis* transport components carrying *α*-galactopyranosyl (*α*-gal) epitopes, a highly immunogenic molecule, which were efficiently recognized by anti-*α*-gal antibodies from patient with PCM [42]. These data showed that the fungal vesicular products might be important serological markers produced during this disease.

The immunomodulatory activity of fungal EVs is still poorly understood.* In vitro* studies have demonstrated that mammalian macrophages can incorporate fungal EVs, resulting in increased levels of both pro- and anti-inflammatory cytokines [150, 151]. Specifically, in* C. neoformans*, the exposure of macrophages to EVs resulted in their internalization and production of IL-10, TGF-*β*, and TNF-*α*, while for* C. albicans*, the production of IL-10, IL-12, and TGF-*β* was observed. In both studies, fungal EVs stimulated murine macrophages to produce higher levels of NO [150, 151]. This effect probably occurred due to the fungal EVs preparations, which were composed of heterogeneous populations of different size and probably content [148, 150].* M. sympodialis* releases EVs carrying allergen, which induce high levels of TNF-*α* and IL-4, suggesting that vesicles have multiple immunoregulatory functions in atopic eczema. Despite this controversy in host immune response, fungal EVs were capable of stimulating a protective response against infection. Recently, Vargas et al. [151] showed that inoculation of* Galleria mellonella*, a larvae model, with EVs followed by challenge with* C. albicans* reduced the number of recovered viable yeasts in comparison to infected larvae control. Moreover, these authors also observed immunomodulation of DC after internalization of EVs from* C. albicans*. The synthesis of IL-12, IL-10, TGF-*β*, and TNF-*α* was also significantly increased in comparison to nonstimulated DC [151].

Proteomic-based approaches have been used to characterize* C. neoformans*,* P. brasiliensis*,* H. capsulatum*, and* C. albicans* and* S. cerevisiae* EVs [78, 150, 151, 153, 156]. Interestingly, most of the identified proteins in* P. brasiliensis* and* C. neoformans* lacked the characteristic signal peptide required for conventional secretion [78, 156], suggesting that fungal vesicles can also be derived from unconventional secretory mechanisms, as observed in mammalian cells [173]. These proteomic analyses also revealed a large complexity of proteins with diverse biological functions in fungi EVs. Remarkably, we notice the presence of four proteins repeated in all EVs analyzed as follows: glyceraldehyde-3-phosphate dehydrogenase (GADPH), phosphoglycerate kinase, elongation factor 1-alpha, and 6-phosphogluconate dehydrogenase. Thus, it is possible to consider the potential of these molecules as biomarkers of fungal EVs.

## 6. Concluding Remarks

EVs are remarkable structures found in all biological fluids in mammals. The major reported functions of EVs are highlighted in Figure 1. In normal and tumor cells, they affect the following: antigen presentation, immune suppression, intercellular communication, inflammation, cellular homeostasis, and coagulation. In pathogens, they are considered virulence factors and are involved in the following: cell adhesion and invasion, evasion and modulation of the immune response, and drug resistance.

There are many molecules in EVs. The EVs from mammalian cells contain molecules such as MHC classes I and II, mRNA, miRNA, caspase 3, signaling factors, structural proteins, and cytokines. The EVs isolated from tumor cells express, for example, FasL, MHC classes I and II, mRNA, miRNA, FADD, P-glycoprotein, MMPs, PS, and TF. In protozoan, EVs are formed by key membrane components involved in host-parasite interaction. OMV or EVs from bacteria have antigenic material providing gene transference of resistance to antibiotics and adaptation factors. Fungal EVs are structures for transcell transport of virulence factors, immunomodulatory molecules, and serological markers. Therefore, EVs extend the cell-to-cell communication between host and pathogens. By preventing this communication, EVs can be used as targets for vaccination. In addition, the presence of EVs and the characterization of their composition can provide new diagnostic information on several diseases. Furthermore, studies on EVs in the different situations can be useful to understand the intimate mechanisms of pathogenesis. In conclusion, EVs represent a rich and challenging subject for basic and applied research enabling the understanding of a plethora of different mechanisms and opening new tools to combat diseases (Figure 2).

## Figures and Tables

**Table 1:** Major components of extracellular vesicles and their functions described.

Origin	Molecule	Function	Reference
Tumor	MHC I and II	Antigen presentation	[18]
	miRNA and mRNA	Oncogenic activity, drug resistance, and metastasis angiogenesis	[19–22]
	CXCR4 and MMP-9	Invasion and migration	[23]
	TrkB, EGFR, and TES complex	Angiogenesis	[24]
	Rab22A, Pabp1, and PSA	Metastasis	[25–27]
	CD40, CD80, CD86, and CD54	Immunity	[28]
	GTPase and Rab27a	Upregulated immune system and inhibited tumor growth	[29]

Bacteria	OmpQ and pertactin	Immunogenic	[30]
	Gene transfer	Communication	[31]
	Gentamycin	Cell death	[32]
	RNAs	Communication	[33, 34]

Protozoa	tGPI-mucin	Activation	[35]
	Tc85	Invasion/adhesion	[36]
	gp63 and LPG	Virulence factor	[37–39]
	TS	Virulence factor	[40, 41]

Fungi	*α*-gal	Immunogenic	[42]
	GXM and GlcCer	Virulence factor	[43–45]

Eukaryotic mammalian cells	MHC I and II	Antigen presentation	[2]
	FasL	Immune suppression	[13]

## Abbreviations

ALIX: ALG-2-interacting protein X
AP-1: Activator protein 1
CD55: Complement decay-accelerating factor
EGFR: Epidermal growth factor receptor
FADD: Fas-associated protein with death domain
FasL: Fas ligand
HSPs: Heat shock proteins
MHC: Major histocompatibility complex
MyD88: Myeloid differentiation primary response gene (88)
NF-kB: Nuclear factor kappa-light-chain-enhancer of activated B cells
RAB GTPases: Ras-related in brain GTPases
TGF-*β*: Transforming growth factor beta
TLR4: Toll-like receptor 4
TNF-*α*: Tumor necrosis factor
TrkB: Tropomyosin related kinase B
TSG101: Tumor susceptibility gene 101.

## References

[1] Kourembanas S. (2015). Exosomes: vehicles of intercellular signaling, biomarkers, and vectors of cell therapy. *Annual Review of Physiology*.

[2] Théry C., Ostrowski M., Segura E. (2009). Membrane vesicles as conveyors of immune responses. *Nature Reviews Immunology*.

[3] Théry C., Zitvogel L., Amigorena S. (2002). Exosomes: Composition, biogenesis and function. *Nature Reviews Immunology*.

[4] Colombo M., Raposo G., Théry C. (2014). Biogenesis, secretion, and intercellular interactions of exosomes and other extracellular vesicles. *Annual Review of Cell and Developmental Biology*.

[5] Deatheragea B. L., Cooksona B. T. (2012). Membrane vesicle release in bacteria, eukaryotes, and archaea: a conserved yet underappreciated aspect of microbial life. *Infection and Immunity*.

[6] le Pecq J. B. (2005). Dexosomes as a therapeutic cancer vaccine: from bench to bedside. *Blood Cells, Molecules, and Diseases*.

[7] Stegmayr B., Ronquist G. (1982). Promotive effect on human sperm progressive motility by prostasomes. *Urological Research*.

[8] Tanimura A., McGregor D. H., Anderson H. C. (1983). Matrix vesicles in atherosclerotic calcification. *Proceedings of the Society for Experimental Biology and Medicine*.

[9] Mollard G. F. V., Mignery G. A., Baumert M. (1990). rab3 is a small GTP-binding protein exclusively localized to synaptic vesicles. *Proceedings of the National Academy of Sciences of the United States of America*.

[10] Mathivanan S., Ji H., Simpson R. J. (2010). Exosomes: extracellular organelles important in intercellular communication. *Journal of Proteomics*.

[11] Andreu Z., Yanez-Mo M. (2014). Tetraspanins in extracellular vesicle formation and function. *Frontiers in Immunology*.

[12] Mathivanan S., Fahner C. J., Reid G. E., Simpson R. J. (2012). ExoCarta 2012: database of exosomal proteins, RNA and lipids. *Nucleic Acids Research*.

[13] Martínez-Lorenzo M. J., Anel A., Gamen S. (1999). Activated human T cells release bioactive Fas ligand and APO2 ligand in microvesicles. *Journal of Immunology*.

[14] Germain S. J., Sacks G. P., Soorana S. R., Sargent I. L., Redman C. W. (2007). Systemic inflammatory priming in normal pregnancy and preeclampsia: the role of circulating syncytiotrophoblast microparticles. *Journal of Immunology*.

[15] Messerli M., May K., Hansson S. R. (2010). Feto-maternal interactions in pregnancies: placental microparticles activate peripheral blood monocytes. *Placenta*.

[16] Al-Nedawi K., Meehan B., Micallef J. (2008). Intercellular transfer of the oncogenic receptor EGFRvIII by microvesicles derived from tumour cells. *Nature Cell Biology*.

[17] Higginbotham J. N., Demory Beckler M., Gephart J. D. (2011). Amphiregulin exosomes increase cancer cell invasion. *Current Biology*.

[18] Théry C., Duban L., Segura E., Væron P., Lantz O., Amigorena S. (2002). Indirect activation of naïve CD4^+^ T cells by dendritic cell-derived exosomes. *Nature Immunology*.

[19] Wei Y., Lai X., Yu S. (2014). Exosomal miR-221/222 enhances tamoxifen resistance in recipient ER-positive breast cancer cells. *Breast Cancer Research and Treatment*.

[20] Zhou W., Fong M. Y., Min Y. (2014). Cancer-secreted miR-105 destroys vascular endothelial barriers to promote metastasis. *Cancer Cell*.

[21] Wang M., Zhao C., Shi H. (2014). Deregulated microRNAs in gastric cancer tissue-derived mesenchymal stem cells: novel biomarkers and a mechanism for gastric cancer. *British Journal of Cancer*.

[22] Valencia K., Luis-Ravelo D., Bovy N. (2014). MiRNA cargo within exosome-like vesicle transfer influences metastatic bone colonization. *Molecular Oncology*.

[23] Chen G., Zhang Y., Wu X. (2014). 786-0 renal cancer cell line-derived exosomes promote 786-0 cell migration and invasion in vitro. *Oncology Letters*.

[24] Wilson C. M., Naves T., Vincent F. (2014). Sortilin mediates the release and transfer of exosomes in concert with two tyrosine kinase receptors. *Journal of Cell Science*.

[25] Wang T., Gilkes D. M., Takano N. (2014). Hypoxia-inducible factors and RAB22A mediate formation of microvesicles that stimulate breast cancer invasion and metastasis. *Proceedings of the National Academy of Sciences of the United States of America*.

[26] Ohshima K., Kanto K., Hatakeyama K. (2014). Exosome-mediated extracellular release of polyadenylate-binding protein 1 in human metastatic duodenal cancer cells. *Proteomics*.

[27] Liu T., Mendes D. E., Berkman C. E. (2014). Functional prostate-specific membrane antigen is enriched in exosomes from prostate cancer cells. *International Journal of Oncology*.

[28] Viaud S., Ploix S., Lapierre V. (2011). Updated technology to produce highly immunogenic dendritic cell-derived exosomes of clinical grade: a critical role of interferon-*γ*. *Journal of Immunotherapy*.

[29] Li W., Mu D., Tian F. (2013). Exosomes derived from Rab27aoverexpressing tumor cells elicit efficient induction of antitumor immunity. *Molecular Medicine Reports*.

[30] Bottero D., Gaillard M. E., Errea A. (2013). Outer membrane vesicles derived from Bordetella parapertussis as an acellular vaccine against Bordetella parapertussis and Bordetella pertussis infection. *Vaccine*.

[31] Yaron S., Kolling G. L., Simon L., Matthews K. R. (2000). Vesicle-mediated transfer of virulence genes from Escherichia coli O157:H7 to other enteric bacteria. *Applied and Environmental Microbiology*.

[32] Kadurugamuwa J. L., Beveridge T. J. (1995). Virulence factors are released from *Pseudomonas aeruginosa* in association with membrane vesicles during normal growth and exposure to gentamicin: a novel mechanism of enzyme secretion. *Journal of Bacteriology*.

[33] Kulkarni H. M., Jagannadham M. V. (2014). Biogenesis and multifaceted roles of outer membrane vesicles from Gram-negative bacteria. *Microbiology*.

[34] Lee E.-Y., Choi D.-S., Kim K.-P., Gho Y. S. (2008). Proteomics in gram-negative bacterial outer membrane vesicles. *Mass Spectrometry Reviews*.

[35] Almeida I. C., Gazzinelli R. T. (2001). Proinflammatory activity of glycosylphosphatidylinositol anchors derived from *Trypanosoma cruzi*: structural and functional analyses. *Journal of Leukocyte Biology*.

[36] Magdesian M. H., Giordano R., Ulrich H. (2001). Infection by *Trypanosoma cruzi*: identification of a parasite ligand and its host cell receptor. *The Journal of Biological Chemistry*.

[37] Silverman J. M., Clos J., Horakova E. (2010). *Leishmania* exosomes modulate innate and adaptive immune responses through effects on monocytes and dendritic cells. *The Journal of Immunology*.

[38] Silverman J. M., Reiner N. E. (2011). *Leishmania* exosomes deliver preemptive strikes to create an environment permissive for early infection. *Frontiers in Cellular and Infection Microbiology*.

[39] de Assis R. R., Ibraim I. C., Nogueira P. M., Soares R. P., Turco S. J. (2012). Glycoconjugates in New World species of *Leishmania*: polymorphisms in lipophosphoglycan and glycoinositolphospholipids and interaction with hosts. *Biochimica et Biophysica Acta—General Subjects*.

[40] Goncalves M. F., Umezawa E. S., Katzin A. M. (1991). *Trypanosoma cruzi*: shedding of surface antigens as membrane vesicles. *Experimental Parasitology*.

[41] Trocoli Torrecilhas A. C., Tonelli R. R., Pavanelli W. R. (2009). *Trypanosoma cruzi*: parasite shed vesicles increase heart parasitism and generate an intense inflammatory response. *Microbes and Infection*.

[42] Vallejo M. C., Matsuo A. L., Ganiko L. (2011). The pathogenic fungus *Paracoccidioides brasiliensis* exports extracellular vesicles containing highly Immunogenic *α*-galactosyl epitopes. *Eukaryotic Cell*.

[43] Zaragoza O., Rodrigues M. L., De Jesus M., Frases S., Dadachova E., Casadevall A. (2009). The capsule of the fungal pathogen *Cryptococcus neoformans*. *Advances in Applied Microbiology*.

[44] Fonseca F. L., Nohara L. L., Cordero R. J. B. (2010). Immunomodulatory effects of serotype B glucuronoxylomannan from *Cryptococcus gattii* correlate with polysaccharide diameter. *Infection and Immunity*.

[45] Rodrigues M. L., Nimrichter L., Cordero R. J., Casadevall A. (2011). Fungal polysaccharides: biological activity beyond the usual structural properties. *Frontiers in Microbiology*.

[46] Atay S., Banskota S., Crow J., Sethi G., Rink L., Godwin A. K. (2014). Oncogenic KIT-containing exosomes increase gastrointestinal stromal tumor cell invasion. *Proceedings of the National Academy of Sciences of the United States of America*.

[47] Chen W.-X., Cai Y.-Q., Lv M.-M. (2014). Exosomes from docetaxel-resistant breast cancer cells alter chemosensitivity by delivering microRNAs. *Tumor Biology*.

[48] Xiao X., Yu S., Li S. (2014). Exosomes: decreased sensitivity of lung cancer A549 cells to cisplatin. *PLoS ONE*.

[49] Corcoran C., Rani S., O'Driscoll L. (2014). miR-34a is an intracellular and exosomal predictive biomarker for response to docetaxel with clinical relevance to prostate cancer progression. *The Prostate*.

[50] Takahashi K., Yan I. K., Kogure T., Haga H., Patel T. (2014). Extracellular vesicle-mediated transfer of long non-coding RNA ROR modulates chemosensitivity in human hepatocellular cancer. *FEBS Open Bio*.

[51] Chen T., Guo J., Yang M., Zhu X., Cao X. (2011). Chemokine-containing exosomes are released from heat-stressed tumor cells via lipid raft-dependent pathway and act as efficient tumor vaccine. *Journal of Immunology*.

[52] Dutta S., Warshall C., Bandyopadhyay C., Dutta D., Chandran B. (2014). Interactions between exosomes from breast cancer cells and primary mammary epithelial cells leads to generation of reactive oxygen species which induce DNA damage response, stabilization of p53 and autophagy in epithelial cells. *PLoS ONE*.

[53] Liu J., Sun H., Wang X. (2014). Increased exosomal microRNA-21 and microRNA-146a levels in the cervicovaginal lavage specimens of patients with cervical cancer. *International Journal of Molecular Sciences*.

[54] Jiang Y. J., Bikle D. D. (2014). LncRNA: a new player in 1alpha, 25(OH)_2_ vitamin D_3_/VDR protection against skin cancer formation. *Experimental Dermatology*.

[55] Kahlert C., Melo S. A., Protopopov A. (2014). Identification of doublestranded genomic dna spanning all chromosomes with mutated KRAS and P53 DNA in the serum exosomes of patients with pancreatic cancer. *Journal of Biological Chemistry*.

[56] Smyth T. J., Redzic J. S., Graner M. W., Anchordoquy T. J. (2014). Examination of the specificity of tumor cell derived exosomes with tumor cells in vitro. *Biochimica et Biophysica Acta—Biomembranes*.

[57] Shimbo K., Miyaki S., Ishitobi H. (2014). Exosome-formed synthetic microRNA-143 is transferred to osteosarcoma cells and inhibits their migration. *Biochemical and Biophysical Research Communications*.

[58] Riches A., Campbell E., Borger E., Powis S. (2014). Regulation of exosome release from mammary epithelial and breast cancer cells—a new regulatory pathway. *European Journal of Cancer*.

[59] Hosseini H. M., Fooladi A. A. I., Soleimanirad J., Nourani M. R., Davaran S., Mahdavi M. (2014). Staphylococcal entorotoxin B anchored exosome induces apoptosis in negative esterogen receptor breast cancer cells. *Tumor Biology*.

[60] Mahmoodzadeh Hosseini H., Ali Imani Fooladi A., Soleimanirad J., Reza Nourani M., Mahdavi M. (2014). Exosome/staphylococcal enterotoxin B, an anti tumor compound against pancreatic cancer. *Journal of B.U.ON.*.

[61] Taylor D. D., Gercel-Taylor C. (2013). The origin, function, and diagnostic potential of RNA within extracellular vesicles present in human biological fluids. *Frontiers in Genetics*.

[62] Yao Y., Chen L., Wei W., Deng X., Ma L., Hao S. (2013). Tumor cell-derived exosome-targeted dendritic cells stimulate stronger CD8^+^ CTL responses and antitumor immunities. *Biochemical and Biophysical Research Communications*.

[63] Näslund T. I., Gehrmann U., Qazi K. R., Karlsson M. C. I., Gabrielsson S. (2013). Dendritic cell-derived exosomes need to activate both T and B Cells to induce antitumor immunity. *Journal of Immunology*.

[64] Näslund T. I., Gehrmann U., Gabrielsson S. (2013). Cancer immunotherapy with exosomes requires B-cell activation. *OncoImmunology*.

[65] Hsu D.-H., Paz P., Villaflor G. (2003). Exosomes as a tumor vaccine: enhancing potency through direct loading of antigenic peptides. *Journal of Immunotherapy*.

[66] Sedlik C., Vigneron J., Torrieri-Dramard L. (2014). Different immunogenicity but similar antitumor efficacy of two DNA vaccines coding for an antigen secreted in different membrane vesicle-associated forms. *Journal of Extracellular Vesicles*.

[67] Baker J. L., Chen L., Rosenthal J. A., Putnam D., DeLisa M. P. (2014). Microbial biosynthesis of designer outer membrane vesicles. *Current Opinion in Biotechnology*.

[68] Mayrand D., Grenier D. (1989). Biological activities of outer membrane vesicles. *Canadian Journal of Microbiology*.

[69] Manning A. J., Kuehn M. J. (2013). Functional advantages conferred by extracellular prokaryotic membrane vesicles. *Journal of Molecular Microbiology and Biotechnology*.

[70] Dorward D. W., Garon C. F. (1990). DNA is packaged within membrane-derived vesicles of gram-negative but not gram-positive bacteria. *Applied and Environmental Microbiology*.

[71] Lee E.-Y., Choi D.-Y., Kim D.-K. (2009). Gram-positive bacteria produce membrane vesicles: proteomics-based characterization of *Staphylococcus aureus*-derived membrane vesicles. *Proteomics*.

[72] Kim S.-H., Kim K.-S., Lee S.-R. (2009). Structural modifications of outer membrane vesicles to refine them as vaccine delivery vehicles. *Biochimica et Biophysica Acta—Biomembranes*.

[73] Kesty N. C., Kuehn M. J. (2004). Incorporation of heterologous outer membrane and periplasmic proteins into *Escherichia coli* outer membrane vesicles. *The Journal of Biological Chemistry*.

[74] Kesty N. C., Mason K. M., Reedy M., Miller S. E., Kuehn M. J. (2004). Enterotoxigenic *Escherichia coli* vesicles target toxin delivery into mammalian cells. *The EMBO Journal*.

[75] Bauman S. J., Kuehn M. J. (2009). *Pseudomonas aeruginosa* vesicles associate with and are internalized by human lung epithelial cells. *BMC Microbiology*.

[76] Bauman S. J., Kuehn M. J. (2006). Purification of outer membrane vesicles from *Pseudomonas aeruginosa* and their activation of an IL-8 response. *Microbes and Infection*.

[77] Fiocca R., Necchi V., Sommi P. (1999). Release of *Helicobacter pylori* vacuolating cytotoxin by both a specific secretion pathway and budding of outer membrane vesicles. Uptake of released toxin and vesicles by gastric epithelium. *The Journal of Pathology*.

[78] Rodrigues M. L., Nakayasu E. S., Oliveira D. L. (2008). Extracellular vesicles produced by Cryptococcus neoformans contain protein components associated with virulence. *Eukaryotic Cell*.

[79] Rodrigues M. L., Nimrichter L., Oliveira D. L. (2007). Vesicular polysaccharide export in *Cryptococcus neoformans* is a eukaryotic solution to the problem of fungal trans-cell wall transport. *Eukaryotic Cell*.

[80] Kulp A., Kuehn M. J. (2010). Biological Functions and biogenesis of secreted bacterial outer membrane vesicles. *Annual Review of Microbiology*.

[81] van der Pol E., Böing A. N., Harrison P., Sturk A., Nieuwland R. (2012). Classification, functions, and clinical relevance of extracellular vesicles. *Pharmacological Reviews*.

[82] Acevedo R., Fernández S., Zayas C. (2014). Bacterial outer membrane vesicles and vaccine applications. *Frontiers in Immunology*.

[83] Rivera J., Cordero R. J. B., Nakouzi A. S., Frases S., Nicola A., Casadevall A. (2010). Bacillus anthracis produces membrane-derived vesicles containing biologically active toxins. *Proceedings of the National Academy of Sciences of the United States of America*.

[84] Mashburn-Warren L., Mclean R. J. C., Whiteley M. (2008). Gram-negative outer membrane vesicles: beyond the cell surface. *Geobiology*.

[85] Choi C. W., Park E. C., Yun S. H. (2014). Proteomic characterization of the outer membrane vesicle of *Pseudomonas putida* KT2440. *Journal of Proteome Research*.

[86] Altindis E., Fu Y., Mekalanos J. J. (2014). Proteomic analysis of *Vibrio cholerae* outer membrane vesicles. *Proceedings of the National Academy of Sciences of the United States of America*.

[87] Kouokam J. C., Wai S. N., Fällman M., Dobrindt U., Hacker J., Uhlin B. E. (2006). Active cytotoxic necrotizing factor 1 associated with outer membrane vesicles from uropathogenic Escherichia coli. *Infection and Immunity*.

[88] Bartolini E., Ianni E., Frigimelica E. (2013). Recombinant outer membrane vesicles carrying *Chlamydia muridarum* HtrA induce antibodies that neutralize chlamydial infection in vitro. *Journal of Extracellular Vesicles*.

[89] Aghasadeghi M. R., Salmani A. S., Sadat S. M. (2011). Application of outer membrane vesicle of Neisseria meningitidis serogroup B as a new adjuvant to induce strongly Th1-oriented responses against HIV-1. *Current HIV Research*.

[90] Schnitzer J. K., Berzel S., Fajardo-Moser M., Remer K. A., Moll H. (2010). Fragments of antigen-loaded dendritic cells (DC) and DC-derived exosomes induce protective immunity against *Leishmania major*. *Vaccine*.

[91] Ramachandra L., Qu Y., Wang Y. (2010). *Mycobacterium tuberculosis* synergizes with ATP to induce release of microvesicles and exosomes containing major histocompatibility complex class II molecules capable of antigen presentation. *Infection and Immunity*.

[92] Moshiri A., Dashtbani-Roozbehani A., Peerayeh S. N., Siadat S. D. (2012). Outer membrane vesicle: a macromolecule with multifunctional activity. *Human Vaccines and Immunotherapeutics*.

[93] Sharpe S. W., Kuehn M. J., Mason K. M. (2011). Elicitation of epithelial cell-derived immune effectors by outer membrane vesicles of nontypeable *Haemophilus influenzae*. *Infection and Immunity*.

[94] Ren D., Nelson K. L., Uchakin P. N., Smith A. L., Gu X.-X., Daines D. A. (2012). Characterization of extended co-culture of non-typeable *Haemophilus influenzae* with primary human respiratory tissues. *Experimental Biology and Medicine*.

[95] Beveridge T. J. (1999). Structures of gram-negative cell walls and their derived membrane vesicles. *Journal of Bacteriology*.

[96] Kuehn M. J., Kesty N. C. (2005). Bacterial outer membrane vesicles and the host-pathogen interaction. *Genes and Development*.

[97] Zhao K., Deng X., He C., Yue B., Wu M. (2013). *Pseudomonas aeruginosa* outer membrane vesicles modulate host immune responses by targeting the toll-like receptor 4 signaling pathway. *Infection and Immunity*.

[98] Deknuydt F., Nordström T., Riesbeck K. (2014). Diversion of the host humoral response: A novel virulence mechanism of Haemophilus influenzae mediated via outer membrane vesicles. *Journal of Leukocyte Biology*.

[99] Rossi O., Pesce I., Giannelli C. (2014). Modulation of endotoxicity of Shigella generalized modules for membrane antigens (GMMA) by genetic lipid A modifications: relative activation of TLR4 and TLR2 pathways in different mutants. *The Journal of Biological Chemistry*.

[100] Freixeiro P., Diéguez-Casal E., Costoya L. (2013). Study of the stability of proteoliposomes as vehicles for vaccines against *Neisseria meningitidis* based on recombinant porin complexes. *International Journal of Pharmaceutics*.

[101] Delbos V., Lemée L., Bénichou J. (2013). Impact of MenBvac, an outer membrane vesicle (OMV) vaccine, on the meningococcal carriage. *Vaccine*.

[102] Sjursen H., Wedege E., Rosenqvist E. (1990). IgG subclass antibodies to serogroup B meningococcal outer membrane antigens following infection and vaccination. *APMIS*.

[103] Holst J., Oster P., Arnold R. (2013). Vaccines against meningococcal serogroup B disease containing outer membrane vesicles (OMV): lessons from past programs and implications for the future. *Human Vaccines and Immunotherapeutics*.

[104] Bjune G., Hoiby E. A., Gronnesby J. K. (1991). Effect of outer membrane vesicle vaccine against group B meningococcal disease in Norway. *The Lancet*.

[105] Sadarangani M., Pollard A. J. (2010). Serogroup B meningococcal vaccines-an unfinished story. *The Lancet Infectious Diseases*.

[106] Caron F., du Châtelet I. P., Leroy J.-P. (2011). From tailor-made to ready-to-wear meningococcal B vaccines: longitudinal study of a clonal meningococcal B outbreak. *The Lancet Infectious Diseases*.

[107] van der Ley P., van den Dobbelsteen G. (2011). Next-generation outer membrane vesicle vaccines against *Neisseria meningitidis* based on nontoxic LPS mutants. *Human Vaccines*.

[108] Kim O. Y., Hong B. S., Park K.-S. (2013). Immunization with *Escherichia coli* outer membrane vesicles protects bacteria-induced lethality via Th1 and Th17 cell responses. *The Journal of Immunology*.

[109] Rodríguez-Ortega M. J., Norais N., Bensi G. (2006). Characterization and identification of vaccine candidate proteins through analysis of the group A *Streptococcus* surface proteome. *Nature Biotechnology*.

[110] Kim M.-R., Hong S.-W., Choi E.-B. (2012). *Staphylococcus aureus*-derived extracellular vesicles induce neutrophilic pulmonary inflammation via both Th1 and Th17 cell responses. *Allergy*.

[111] Marcilla A., Martin-Jaular L., Trelis M. (2014). Extracellular vesicles in parasitic diseases. *Journal of Extracellular Vesicles*.

[112] Cronemberger-Andrade A., Aragão-França L., de Araujo C. F. (2014). Extracellular vesicles from leishmania-infected macrophages confer an anti-infection cytokine-production profile to naïve macrophages. *PLoS Neglected Tropical Diseases*.

[113] Bayer-Santos E., Aguilar-Bonavides C., Rodrigues S. P. (2013). Proteomic analysis of trypanosoma cruzi secretome: characterization of two populations of extracellular vesicles and soluble proteins. *Journal of Proteome Research*.

[114] Torrecilhas A. C., Schumacher R. I., Alves M. J. M., Colli W. (2012). Vesicles as carriers of virulence factors in parasitic protozoan diseases. *Microbes and Infection*.

[115] Cestari I., Ansa-Addo E., Deolindo P., Inal J. M., Ramirez M. I. (2012). Trypanosoma cruzi immune evasion mediated by host cell-derived microvesicles. *Journal of Immunology*.

[116] Geiger A., Hirtz C., Bécue T. (2010). Exocytosis and protein secretion in *Trypanosoma*. *BMC Microbiology*.

[117] Campos F. M., Franklin B. S., Teixeira-Carvalho A. (2010). Augmented plasma microparticles during acute *Plasmodium vivax* infection. *Malaria Journal*.

[118] Mantel P. Y., Hoang A. N., Goldowitz I. (2013). Malaria-infected erythrocyte-derived microvesicles mediate cellular communication within the parasite population and with the host immune system. *Cell Host and Microbe*.

[119] Twu O., de Miguel N., Lustig G. (2013). *Trichomonas vaginalis* exosomes deliver cargo to host cells and mediate host:parasite
interactions. *PLoS Pathogens*.

[120] Bhatnagar S., Shinagawa K., Castellino F. J., Schorey J. S. (2007). Exosomes released from macrophages infected with intracellular pathogens stimulate a proinflammatory response in vitro and in vivo. *Blood*.

[121] Beauvillain C., Ruiz S., Guiton R., Bout D., Dimier-Poisson I. (2007). A vaccine based on exosomes secreted by a dendritic cell line confers protection against *T. gondii* infection in syngeneic and allogeneic mice. *Microbes and Infection*.

[122] Beauvillain C., Juste M. O., Dion S., Pierre J., Dimier-Poisson I. (2009). Exosomes are an effective vaccine against congenital toxoplasmosis in mice. *Vaccine*.

[123] del Cacho E., Gallego M., Lee S. H. (2012). Induction of protective immunity against *Eimeria tenella*, *Eimeria maxima*, and *Eimeria acervulina* infections using dendritic cell-derived exosomes. *Infection and Immunity*.

[124] Bernal D., Trelis M., Montaner S. (2014). Surface analysis of *Dicrocoelium dendriticum*. The molecular characterization of exosomes reveals the presence of miRNAs. *Journal of Proteomics*.

[125] Coura J. R. (2013). Chagas disease: control, elimination and eradication. Is it possible?. *Memorias do Instituto Oswaldo Cruz*.

[126] Almeida I. C., Camargo M. M., Procópio D. O. (2000). Highly purified glycosylphosphatidylinositols from *Trypanosoma cruzi* are potent proinflammatory agents. *The EMBO Journal*.

[127] Ferguson M. A. J. (1999). The structure, biosynthesis and functions of glycosylphosphatidylinositol anchors, and the contributions of trypanosome research. *Journal of Cell Science*.

[128] Campos M. A. S., Almeida I. C., Takeuchi O. (2001). Activation of toll-like receptor-2 by glycosylphosphatidylinositol anchors from a protozoan parasite. *Journal of Immunology*.

[129] Golgher D., Gazzinelli R. T. (2004). Innate and acquired immunity in the pathogenesis of Chagas disease. *Autoimmunity*.

[130] Soares R. P., Torrecilhas A. C., Assis R. R. (2012). Intraspecies variation in *Trypanosoma cruzi* GPI-mucins: biological activities and differential expression of alpha-galactosyl residues. *The American Journal of Tropical Medicine and Hygiene*.

[131] Almeida I. C., Ferguson M. A. J., Schenkman S., Travassos L. R. (1994). Lytic anti-alpha-galactosyl antibodies from patients with chronic Chagas' disease recognize novel *O*-linked oligosaccharides on mucin-like glycosyl-phosphatidylinositol-anchored glycoproteins of *Trypanosoma cruzi*. *Biochemical Journal*.

[132] Serna C., Lara J. A., Rodrigues S. P., Marques A. F., Almeida I. C., Maldonado R. A. (2014). A synthetic peptide from Trypanosoma cruzi mucin-like associated surface protein as candidate for a vaccine against Chagas disease. *Vaccine*.

[133] Lambertz U., Silverman J. M., Nandan D. (2012). Secreted virulence factors and immune evasion in visceral leishmaniasis. *Journal of Leukocyte Biology*.

[134] Silverman J. M., Reiner N. E. (2011). Exosomes and other microvesicles in infection biology: organelles with unanticipated phenotypes. *Cellular Microbiology*.

[135] Wylie C. E., Carbonell-Antoñanzas M., Aiassa E. (2014). A systematic review of the efficacy of prophylactic control measures for naturally-occurring canine leishmaniosis, part I: vaccinations. *Preventive Veterinary Medicine*.

[136] Twu O., de Miguel N., Lustig G. (2013). Trichomonas vaginalis exosomes deliver cargo to host cells and mediate host: parasite interactions. *PLoS Pathogens*.

[137] Martin-Jaular L., Nakayasu E. S., Ferrer M., Almeida I. C., del Portillo H. A. (2011). Exosomes from *Plasmodium yoelii*-infected reticulocytes protect mice from lethal infections. *PLoS ONE*.

[138] Regev-Rudzki N., Wilson D. W., Carvalho T. G. (2013). Cell-cell communication between malaria-infected red blood cells via exosome-like vesicles. *Cell*.

[139] Hunter C. A., Sibley L. D. (2012). Modulation of innate immunity by *Toxoplasma gondii* virulence effectors. *Nature Reviews Microbiology*.

[140] Quah B. J. C., O'Neill H. C. (2005). The immunogenicity of dendritic cell-derived exosomes. *Blood Cells, Molecules, and Diseases*.

[141] Bourguin I., Moser M., Buzoni-Gatel D. (1998). Murine dendritic cells pulsed *in vitro* with *Toxoplasma gondii* antigens induce protective immunity *in vivo*. *Infection and Immunity*.

[142] Aline F., Bout D., Amigorena S., Roingeard P., Dimier-Poisson I. (2004). Toxoplasma gondii antigen-pulsed-dendritic cell-derived exosomes induce a protective immune response against *T. gondii* infection. *Infection and Immunity*.

[143] del Cacho E., Gallego M., Lee S. H. (2011). Induction of protective immunity against *Eimeria tenella* infection using antigen-loaded dendritic cells (DC) and DC-derived exosomes. *Vaccine*.

[144] del Cacho E., Gallego M., Lillehoj H. S., Quilez J., Lillehoj E. P., Sánchez-Acedo C. (2013). Tetraspanin-3 regulates protective immunity against *Eimeria tenella* infection following immunization with dendritic cell-derived exosomes. *Vaccine*.

[145] Newman S. L., Smulian A. G. (2013). Iron uptake and virulence in *Histoplasma capsulatum*. *Current Opinion in Microbiology*.

[146] Bartha I., Fellay J. (2015). Adaptation on a genomic scale. *eLife*.

[147] Rodrigues M. L., Nakayasu E. S., Almeida I. C., Nimrichter L. (2014). The impact of proteomics on the understanding of functions and biogenesis of fungal extracellular vesicles. *Journal of Proteomics*.

[148] Casadevall A., Nosanchuk J. D., Williamson P., Rodrigues M. L. (2009). Vesicular transport across the fungal cell wall. *Trends in Microbiology*.

[149] Free S. J. (2013). Fungal Cell Wall Organization and Biosynthesis. *Advances in Genetics*.

[150] Oliveira D. L., Freire-de-Lima C. G., Nosanchuk J. D., Casadevall A., Rodrigues M. L., Nimrichter L. (2010). Extracellular vesicles from *Cryptococcus neoformans* modulate macrophage functions. *Infection and Immunity*.

[151] Vargas G., Rocha J. D. B., Oliveira D. L. (2015). Compositional and immunobiological analyses of extracellular vesicles released by *Candida albicans*. *Cellular Microbiology*.

[152] Rodrigues M. L., Shi L., Barreto-Bergter E. (2007). Monoclonal antibody to fungal glucosylceramide protects mice against lethal *Cryptococcus neoformans* infection. *Clinical and Vaccine Immunology*.

[153] Albuquerque P. C., Nakayasu E. S., Rodrigues M. L. (2008). Vesicular transport in Histoplasma capsulatum: an effective mechanism for trans-cell wall transfer of proteins and lipids in ascomycetes. *Cellular Microbiology*.

[154] Oliveira D. L., Nakayasu E. S., Joffe L. S. (2010). Characterization of yeast extracellular vesicles: evidence for the participation of different pathways of cellular traffic in vesicle biogenesis. *PLoS ONE*.

[155] Vallejo M. C., Nakayasu E. S., Longo L. V. G. (2012). Lipidomic analysis of extracellular vesicles from the pathogenic phase of *Paracoccidioides brasiliensis*. *PLoS ONE*.

[156] Vallejo M. C., Nakayasu E. S., Matsuo A. L. (2012). Vesicle and vesicle-free extracellular proteome of *Paracoccidioides brasiliensis*: comparative analysis with other pathogenic fungi. *Journal of Proteome Research*.

[157] Oliveira D. L., Nimrichter L., Miranda K. (2009). Cryptococcus neoformans cryoultramicrotomy and vesicle fractionation reveals an intimate association between membrane lipids and glucuronoxylomannan. *Fungal Genetics and Biology*.

[158] Eisenman H. C., Frases S., Nicola A. M., Rodrigues M. L., Casadevall A. (2009). Vesicle-associated melanization in *Cryptococcus neoformans*. *Microbiology*.

[159] Albuquerque P. C., Cordero R. J. B., Fonseca F. L. (2012). A Paracoccidioides brasiliensis glycan shares serologic and functional properties with cryptococcal glucuronoxylomannan. *Fungal Genetics and Biology*.

[160] Nimrichter L., Rodrigues M. L. (2011). Fungal glucosylceramides: from structural components to biologically active targets of new antimicrobials. *Frontiers in Microbiology*.

[161] Toledo M. S., Suzuki E., Levery S. B., Straus A. H., Takahashi H. K. (2001). Characterization of monoclonal antibody MEST-2 specific to glucosylceramide of fungi and plants. *Glycobiology*.

[162] Rodrigues M. L., Travassos L. R., Miranda K. R. (2000). Human antibodies against a purified glucosylceramide from *Cryptococcus neoformans* inhibit cell budding and fungal growth. *Infection and Immunity*.

[163] Rittershaus P. C., Kechichian T. B., Allegood J. C. (2006). Glucosylceramide synthase is an essential regulator of pathogenicity of *Cryptococcus neoformans*. *The Journal of Clinical Investigation*.

[164] Noble S. M., French S., Kohn L. A., Chen V., Johnson A. D. (2010). Systematic screens of a *Candida albicans* homozygous deletion library decouple morphogenetic switching and pathogenicity. *Nature Genetics*.

[165] Guimaraes L. L., Toledo M. S., Ferreira F. A. S., Straus A. H., Takahashi H. K. (2014). Structural diversity and biological significance of glycosphingolipids in pathogenic and opportunistic fungi. *Frontiers in Cellular and Infection Microbiology*.

[166] Albacker L. A., Chaudhary V., Chang Y.-J. (2013). Invariant natural killer T cells recognize a fungal glycosphingolipid that can induce airway hyperreactivity. *Nature Medicine*.

[167] Garcia-Rivera J., Eisenman H. C., Nosanchuk J. D. (2005). Comparative analysis of *Cryptococcus neoformans* acid-resistant particles generated from pigmented cells grown in different laccase substrates. *Fungal Genetics and Biology*.

[168] Taborda C. P., da Silva M. B., Nosanchuk J. D., Travassos L. R. (2008). Melanin as a virulence factor of *Paracoccidioides brasiliensis* and other dimorphic pathogenic fungi: a minireview. *Mycopathologia*.

[169] Wang Y., Aisen P., Casadevall A. (1995). *Cryptococcus neoformans* melanin and virulence: mechanism of action. *Infection and Immunity*.

[170] Rosas Á. L., Nosanchuk J. D., Feldmesser M., Cox G. M., McDade H. C., Casadevall A. (2000). Synthesis of polymerized melanin by *Cryptococcus neoformans* in infected rodents. *Infection and Immunity*.

[171] da Silva M. B., Marques A. F., Nosanchuk J. D., Casadevall A., Travassos L. R., Taborda C. P. (2006). Melanin in the dimorphic fungal pathogen *Paracoccidioides brasiliensis*: effects on phagocytosis, intracellular resistance and drug susceptibility. *Microbes and Infection*.

[172] Van Duin D., Casadevall A., Nosanchuk J. D. (2002). Melanization of Cryptococcus neoformans and *Histoplasma capsulatum* reduces their susceptibilities to amphotericin B and caspofungin. *Antimicrobial Agents and Chemotherapy*.

[173] Zhang M., Schekman R. (2013). Cell biology: unconventional secretion, unconventional solutions. *Science*.

